# Beyond divest vs. engage: a review of the role of institutional investors in an inclusive fossil fuel phase-out

**DOI:** 10.1080/14693062.2023.2261900

**Published:** 2023-10-12

**Authors:** Clara McDonnell, Joyeeta Gupta

**Affiliations:** a Department of Geography, Planning and International Development, University of Amsterdam’s Governance and Inclusive Development Research Group, Amsterdam, Netherlands; b IHE-Delft Institute for Water Education, Delft, Netherlands

**Keywords:** Institutional investors, pension funds, asset managers, fossil fuels, climate, inclusive development

## Abstract

Institutional investors, who control as much as $154 trillion globally, may play an important role in shaping the energy transition as major stakeholders in fossil fuel producing, distributing and consuming companies. Research on investors and fossil fuels has focused largely on the divestment movement or on shareholder engagement. However, given their limited success to date, additional strategies to influence the fossil fuel sector are merited. This review paper expands the scope of attention to investors, asking: what strategies for influencing the fossil fuel industry are available to institutional investors and what are the implications of these for achieving an inclusive fossil fuel phase-out? Through a systematic review of 153 papers, we identify seven strategies for influencing the fossil fuel phase-out: divestment, shareholder engagement, hiring practices, engaging the financial sector, engaging indirect financial actors, litigation, and green investment. These strategies represent ways for investors to increase the impact of their engagements, as well as areas deserving greater attention from academics, policymakers, and activists. Across these strategies, we note trade-offs in favour of financial returns at the expense of social, ecological, and equity outcomes. We argue that future research should focus on: (a) the role of under-studied actors in aligning finance with climate goals; (b) the implications of investor action for an inclusive energy transition; and (c) policy solutions capable of overcoming investors’ short-term profit motives to instead incentivise long-term investor engagement with climate issues.

## Key policy highlights

- Legal mandates and uncertainties in how to apply fiduciary responsibility with respect to climate change result in investors and asset managers prioritizing short-term profits at the expense of climate goals.
- Voluntary investor efforts focus predominantly on transparency and disclosure and are insufficient to meet climate goals. Legally binding decarbonization strategies are needed to align finance with the Paris Agreement.
- Influence over investment strategies is increasingly concentrated in a small number of powerful actors. Policymakers should consider not only asset owners, but also asset managers, index providers, and proxy advisory firms.
- Investors can increase their climate action by incorporating policies on climate into their mandates for asset managers and engaging with fossil fuel financiers.

## 1. Introduction

Article 2.1c of the 2015 Paris Agreement on Climate Change requires financial flows to be consistent with its goals to limit global warming to 1.5-2℃ and with ‘climate-resilient development pathways.’ Meeting these goals will require a rapid fossil fuel (FF) phase-out and enormous investment into renewable alternatives. By complying with Article 2.1c, institutional investors, who control approximately $154^1^ trillion globally, could play a major role in the energy transition (Thinking Ahead Institute, 2021a). Given their level of influence as major shareholders, and their interest in avoiding financial risks associated with climate change, investors, financial regulators, and other actors in the financial sector may be a pragmatic, if not ideal, ‘second best’ source of climate action when political intervention appears unlikely (Gunningham, 2020). We focus in particular on the influential role that pension funds and asset managers might play in a FF phase-out, although our findings are relevant for other institutional investors.^2^ Pension funds make up the largest category of asset owners; with approximately $57 trillion in assets, they are considered to have an interest in the long-term sustainability of their investments (compared to e.g. hedge funds), and are historically active shareholders (Clark & Hebb, 2004; Thinking Ahead Institute, 2021a). Asset managers are increasingly important, given the rapid growth of the sector and its influence over how assets of other investors are invested (see 3.3.1). To date, scholarship on how investors could influence FF and other highly polluting companies has focused on divestment (selling one’s stake in a company) or shareholder engagement, which encompasses a range of strategies investors use to influence management. Despite growing academic and media attention to both strategies (e.g. Godfrey, 2022), neither has been successful in cutting FF production, as global emissions continue to rise and levels of FF investments remain high (IEA, 2022; IPCC, 2022). This review thus aims to broaden the debate and considers additional avenues through which institutional investors might contribute to phasing out FF.

The Paris Agreement also acknowledges the importance (‘for some’) of climate justice and a ‘just transition’ (UNFCCC, 2015). The private sector, and especially institutional investors, are expected to be critical for meeting the funding needs required to implement the Sustainable Development Goals (SDGs) (estimated to be as much as $3.7 trillion annually (PRI, 2022)). Despite the importance of institutional investors for meeting climate and development goals, there remain many shortfalls and barriers to aligning finance with these goals. The IPCC (2022, p. TS-122) finds that ‘fundamental inequities in access to finance as well as finance terms and conditions, and countries’ exposure to physical impacts of climate change overall, result in a worsening outlook for a global just transition.’ We argue that prioritizing a just energy transition, which limits the worst impacts of climate change and, as the SDGs call for, ‘leaves no one behind’, requires an *inclusive* approach. Sustainable development approaches, which aim to balance social, environmental, and economic outcomes, too often result in trade-offs in which economic outcomes are prioritized above all others (Gupta et al., 2015). Inclusive development, which integrates concerns of justice and equity, thus provides a valuable lens through which to examine the financial sector. It centres on three dimensions: social inclusiveness, which calls for equitable access to and sharing of resources and opportunities; ecological inclusiveness, which requires respecting limits to exploitation of the planet’s resources and sinks; and relational inclusiveness, which examines how power imbalances hinder social and ecological inclusiveness (Gupta et al., 2015). Using inclusive development as a lens to critically review the literature is a departure from the dominant approaches that feature in much work on climate and finance, which tend to centre economic dimensions. In doing so, we hope to highlight key areas which merit further attention from policymakers, activists, and academics working towards a just transition. Thus, this review asks: What strategies for influencing the FF industry are available to institutional investors and what are the implications of these for achieving an inclusive FF phase-out?

Below, we first present our methods (2), then present and discuss the results of our review by strategy: divestment (3.1), shareholder engagement (3.2), hiring practices (3.3), investments in the financial sector (3.4), the influence of indirect actors in the financial sector (3.5), litigation (3.6), and green investments (3.7) before discussing (3.8) and drawing conclusions (4).

## 2. Methods

**Figure 1.**
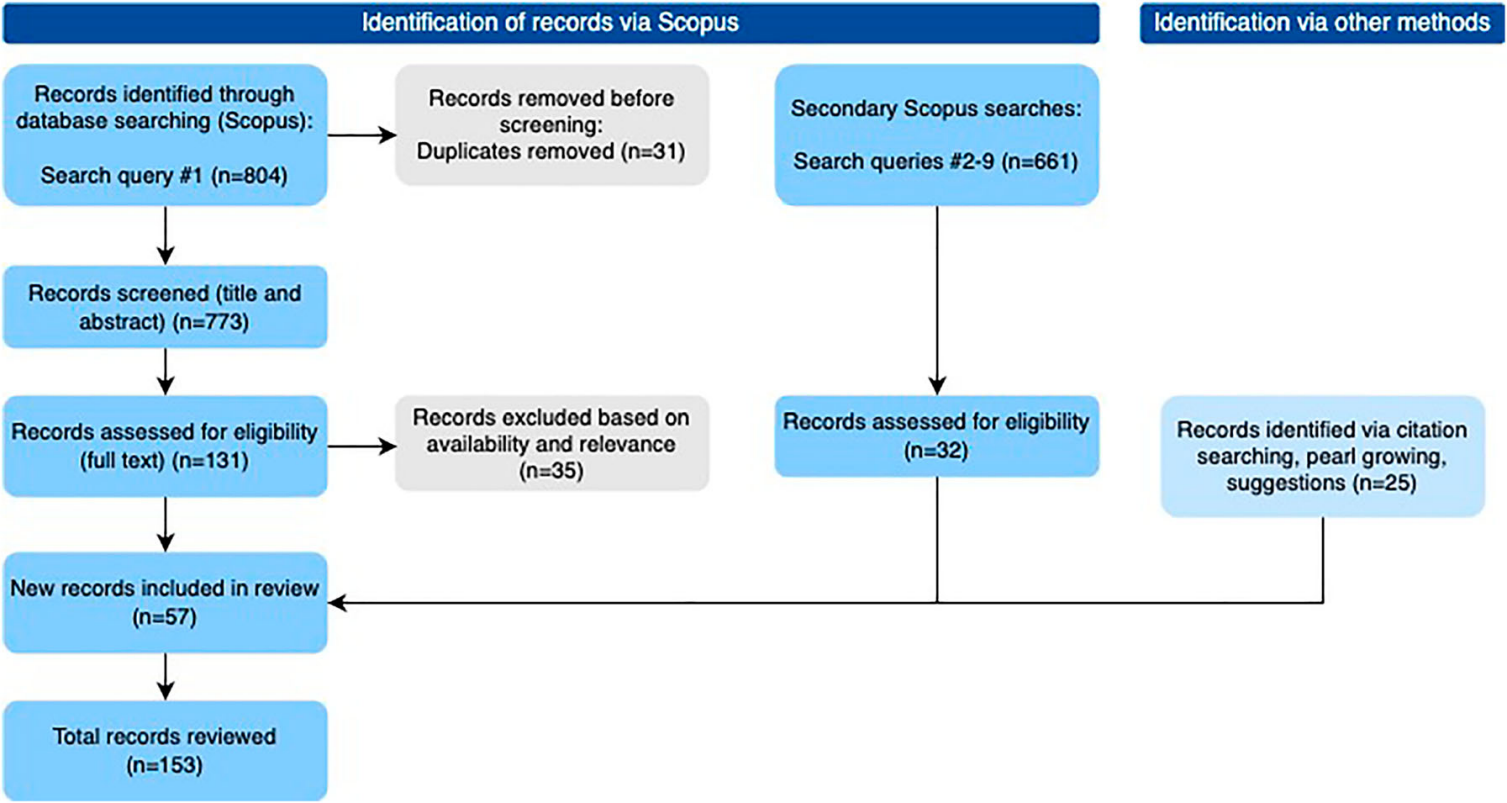
PRISMA diagram, modified from Page et al. (2021).

We employ systematic literature review methods to identify and assess the state of the art of literature available on institutional investors and their role in aligning financial flows with climate change goals and phasing out FF, as well as to identify under-researched areas. Systematic literature reviews aim to produce a comprehensive and transparent synthesis of the body of knowledge available on a particular topic or question (Booth et al., 2016). Our review focuses specifically on the strategies that institutional investors may use to accelerate a FF phase-out. An initial, unstructured, exploration of the literature was first used to gain familiarity with the literature and develop relevant search terms. Given the prominence of pension funds and asset managers among institutional investors, we initially targeted our searches towards these actors alone. However, given the tendency in the literature to refer to ‘institutional investors’ in general, we included this broader category in our searches.^3^ We then conducted a systematic review of the literature available in the Scopus database in two main stages (see Figure 1). First, we review the literature on institutional investors and climate change, or FF specifically. After identifying relevant terms and testing searches to narrow down results to a feasible level, the initial search was conducted: (pension OR ‘institutional investor’ OR ‘asset manager’) AND (climate OR ‘fossil fuel’ OR oil OR coal OR gas), which returned 804 results. Rayyan^4^ was used to screen the titles and abstracts of all returned results. Based on the inclusion criteria (see Table 1) used, 96 journal articles and book chapters were selected for review. During analysis, the articles were coded inductively by investor strategies identified, which enabled us to generate the list of seven strategies discussed in this article. To enrich and verify the state-of-knowledge on each of these strategies, in the second stage, additional targeted searches were conducted for each strategy (see Supplementary Material for complete list). The secondary searches returned a total of 661 results, of which 32 were added to the list of reviewed papers, with the final selection based on the same inclusion criteria developed for the first search. All searches were conducted between August – September 2022. While using the Scopus database has advantages in limiting searches to peer-reviewed literature and sufficiently focusing results to enable a systematic review, we acknowledge that there may be limitations with using one database alone. To minimize these limitations, we ran further searches with our key words as well as relevant synonyms^5^ in both Scopus and Google Scholar to identify any key missing articles. Based on these, as well as through citation searching, pearl-growing methods, and suggested articles^6^, we included a further 25 articles (Papaioannou et al., 2010). Where relevant, we cite grey literature, however, the relevant grey literature was not identified or reviewed systematically.

**Table 1.** Inclusion/Exclusion criteria.

Inclusion Criteria	Exclusion Criteria
Journal articles, book chapters Published in English Relevant to research question, central focus on institutional investors and their actions with respect to any of the following: FF, climate change, sustainability, ESG	Conference proceedings, editorials Alternate understandings of search terms (e.g. refers to ‘political climate’ rather than climate change) Relevance: no investor focus or refers to investment strategies or performance alone with no discussion of climate, sustainability, or FF

## 3. Results

### 3.1. Divestment

Divestment, selling off a type or category of assets, is one of the more well-known strategies for investors to sway the FF industry. Its prominence as a form of climate action is largely due to the work of the global FF divestment movement, spearheaded by the organization 350.org. Since the movement’s emergence, many major financial institutions have made divestment commitments. Specifically, as of September 2022, investors with assets worth $40.48 trillion have committed to divest fully or partially from FFs (Stand.earth, n.d.). Braungardt et al.’s (2019) review of academic and grey literature on divestment presents key arguments for and against divestment (e.g. arguments for include: damaging the ‘social license’ for FF companies to operate, reducing investor exposure to risk, catalysing climate action; and those against: the limited financial effect on FF companies, its focus on FF rather than all emissions). Since Braungardt et al.’s (2019) review, there have been further developments in the academic literature studying FF divestment. Our review identifies 19 articles published since 2019 with FF divestment as their primary focus, with additional articles discussing divestment alongside other strategies. Many of the recent publications are empirical studies, which provide an important addition to Braungardt et al.’s (2019) review, which focuses predominantly on arguments and debates around divestment. We identify three trends in this recent literature: a) predominant interest in the financial implications of divestment; b) an expansion of the concept of divestment; and c) limited attention from scholars on the social and equity implications of divestment.

Studies on the financial implications of divestment have examined the impacts for both investors and for the FF companies being targeted by divestment. While legal arguments for considering FF divestment as compliant with fiduciary duty have been explored (Sarang, 2015; Schneider, 2015), recent studies have tested this claim empirically. Plantinga and Scholtens (2021) test performance of a non-fossil investment portfolio composed of global stocks compared to a FF-inclusive portfolio both over a forty-year period and in modelled future scenarios, finding no significant difference in returns between the two portfolios, in both smooth and delayed projected energy transitions. Al Ayoubi and Enjolras (2022) find similar results for sovereign wealth funds. These findings support and expand on prior studies which modelled returns on portfolios composed of US stocks only (Halcoussis & Lowenberg, 2019; Trinks et al., 2018) or portfolios of Dutch pensions (Boermans & Galema, 2019), and have been supported by reports conducted by the private sector (e.g. Sanzillo, 2021). Evidence of the financial ramifications of divestment for FF companies is limited and mixed. While Dordi and Weber (2019) find some evidence for a short-term stock price drop for the largest FF companies post-prominent divestment announcements, Hansen and Pollin (2020) find minimal evidence of divestment announcements impacting the FF sector (based on the performance of prominent FF indices). Bassen et al.’s (2021) study of BlackRock’s coal divestment found that while most coal companies were not impacted, the share prices of the largest US-based coal companies did experience a significant drop after the divestment announcement. BlackRock’s own share price rose on the day of the announcement, indicating the market’s positive assessment of their decision, and the fact that they sold shares in coal companies prior to announcing divestment, to avoid losses associated with a stock price drop post-divestment announcement.

Though Braungardt et al. (2019) and most previous authors writing about the FF divestment movement discuss divestment in its strictest sense – i.e. the selling off of FF assets (generally stocks and bonds) – recent literature adopts a more expansive definition. Scott (2020) refers to the ‘new ‘divest movement’’ as ‘broadly includ[ing] a variety of activities intended to persuade investment funds, insurers, corporations, and individuals to stop doing business with FF companies.’ (p. 539). Curran’s (2020) case study of the influence of the divestment movement on the Adani’s Carmichael coal mine in Australia employs such a broad definition. In that case, divestment activists targeted not only shareholders and pension funds, but also insurers, banks, and government subsidies. The activists’ goal was not only for large investors to sell off their shares, but also for major financiers to deny additional funding and insurance to Adani for development of the mine. Although the mine was ultimately approved, the tactics of divestment campaigners convinced many financial actors to decline funding the mine, reducing the size of the mine. Cojoianu et al. (2021) and Cojoianu et al. (2021) have innovatively studied the divestment movement by considering its indirect impact on investment banks’ levels of underwriting and loans to the FF sector. This broader understanding of divestment suggests the need for greater nuance in discussions of divestment and distinction between specific strategies, particularly when making arguments for or against divestment.

Finally, we note that literature on investors and FF divestment has focused overwhelmingly on the financial implications of divestment. There is limited engagement with what divestment might mean for creating new vested interests, development opportunities, the distribution of losses associated with stranded assets, and the longer-term operation of FF companies. Rempel and Gupta (2020, 2021) point out that large-scale divestment risks shifting the burden of stranded assets to less powerful financial institutions, potentially in the Global South^7^, to unwary purchasers, or to investors predominantly interested in speculation and short-term profit, merely rearranging the vested interests involved. The transfer of assets to private investors may also reduce the transparency of company operations, limiting the potential for public scrutiny (Christophers, 2021). The Global North–South dynamics and implications of divestment have not yet been examined by empirical research. The divestment research also does not engage with the fact that financial institutions and investors have profited for decades from the production of FFs and enabled further expansion and production through their provision of finance (Rempel & Gupta, 2020). If by divesting their FF assets, investors are able to enjoy the benefits of prior investment while reallocating the burden of stranded assets to others (with potentially less resources at their disposal), this raises significant concerns for a just transition.

### 3.2. Shareholder engagement

The dominant alternative to divestment, shareholder engagement, concerns investors using their role as shareholders with a stake in the company to wield influence over company management. Many institutional investors advocate for shareholder engagement as their preferred strategy, arguing that, compared to divestment, holding onto shares allows them to have ongoing influence over companies (Drollette, 2021; Krueger et al., 2020). Other literature reviews on shareholder engagement and activism have addressed its processes, outcomes, and theoretical approaches (Cundill et al., 2018; Goranova & Ryan, 2014) and the effectiveness of different engagement strategies for achieving social and environmental outcomes (Sjöström, 2020). We focus here particularly on the literature on shareholder engagement on climate change and FF specifically and the barriers preventing engagement from prompting ambitious change within companies.

Shareholder engagement can encompass a wide range of engagement strategies, both public and private. Public forms of shareholder engagement may include investor votes on shareholder resolutions (which are often publicized, or publicly available) or investor press releases and statements to the media. Private forms of shareholder engagement may include meetings or communication between investors and company management. Krueger et al.’s (2020) survey of 439 respondents working within institutional investors identifies the frequency of various investor strategies, finding that most (84%) had conducted some form of climate-related shareholder engagement in the last 5 years, most frequently through discussions with management (43%), and least often through divestment, in the case of unsuccessful engagements (20%). Although most (71%) reported that companies respond to their engagement, this response is generally limited to acknowledgement of the concern. Only 25% of respondents reported that engagements are typically successfully resolved. When unsuccessful, many investors decided to take no further action (40%), while only 21% continued engaging. Shareholder engagement is increasingly being conducted through investor participation in collective investor initiatives, such as Climate Action 100+, the Investor Agenda, and various net-zero initiatives, among others. Though these initiatives have targeted the FF industry, the progress they have made in those engagements has been limited by their voluntary, non-binding nature and minimal requirements for investors (McDonnell et al., 2022).

When it comes to the *content* of shareholder engagement on climate issues, the literature focuses on disclosure, asking companies to provide information on their environmental performance, carbon emissions, or climate risks. Various studies have looked at the role of the CDP (formerly, Carbon Disclosure Project), one of the earlier disclosure-oriented collective investor initiatives which aims to increase company disclosure of their emissions by uniting a broad base of investors to pressure companies to comply with disclosure requests. Through the CDP, investor efforts have effectively increased climate disclosures from companies, though there were certain limitations to their success, including variable quality and comparability of company disclosures and the limited potential for disclosure to indicate future behavioural change from companies (Cotter & Najah, 2012; Haque & Islam, 2015; Sullivan & Gouldson, 2012). Recent studies provide further evidence of the success of institutional investors in increasing company disclosures through their shareholder engagement, also arguing that disclosure can be financially beneficial for companies (Flammer et al., 2021; Kordsachia et al., 2021). Though the quality and consistency of disclosures remains an issue, Ilhan et al. (2021) find that through government action (e.g. France’s implementation of Article 173 of the Energy Transition for Green Growth Act, which requires investors to disclose climate risks) and the efforts of major investor initiatives like Climate Action 100+, the level and quality of climate risk disclosure from companies that French investors invest in have both increased.

However, many authors are sceptical of the potential for disclosure alone to result in meaningful climate action. Disclosure is relatively easy, especially compared to the difficult and costly actions that may be necessary in the energy transition. Disclosure, in itself, will not generate the emissions reductions and changes to financial flows needed (Ameli et al., 2020; Harmes, 2011). Gunningham (2020, p. 7) worries that the dominant focus on disclosure may ‘lull’ policymakers into a ‘mistaken sense that information disclosure and risk management is *all* that is necessary to enable financial actors to play their part in a low carbon transition.’ The translation of disclosure into reduced emissions is not straightforward. Bolton and Kacperczyk (2021) find that most emissions reduction commitments come from companies who have lower emissions to begin with and have a much less difficult pathway for reducing emissions: ‘the movements to get companies to commit have been successful in drawing in the willing but have found greater resistance from the companies that need to reduce their emissions the most’ (Bolton & Kacperczyk, 2021, p. 1).

Despite growing investor attention to engaging with companies on climate, investors’ primary focus remains the threat that climate risks pose to the profitability of their investments (Christophers, 2019; Puri, 2019). While not unexpected, it raises significant concerns if one expects investors to play a major role in driving the energy transition. As many have pointed out, the timeframes against which investors (even long-term investors) measure their financial performances and risks are incredibly short, compared to the timelines on which the effects of climate change are experienced (Ameli et al., 2020; Christophers, 2019; Clark & Hebb, 2004). The urgency and speed of action needed to mitigate the worst climate disasters do not translate, even through disclosure, to the types of immediate economic risks that might spur more ambitious investor action. The current model of shareholder engagement, which calls for company climate action while protecting financial returns may not be sufficient for achieving the types of transformations needed to meet climate goals. Though investors have made progress in introducing climate concerns at companies, the literature often does not engage critically with the scale of investor action or progress. Researchers could demonstrate greater attention to contextualizing this progress within the scale of action needed to meet climate goals.

### 3.3. Hiring practices

The literature indicates that asset managers and investment consultants, which institutional investors hire to advise on or take over the management of investment portfolios, may greatly influence both investments and engagements. Asset managers take on many of the shareholder rights of investors, granting them enormous influence within companies, while consultants may direct investors on investments and selection of asset managers.

#### 3.3.1. Asset managers

Although the literature on investors and climate change tends to focus on long-term institutional investors, primarily pension funds, there is an increasing awareness of the role of asset managers in driving corporate governance. The asset management industry began to take off in the 1980s, when institutional investors began delegating management of their funds to for-profit managers (Braun, 2020). Since the early 2000s, and especially post 2008, the asset management industry has rapidly expanded in size and consolidated, as investors were attracted to the low fees associated with passive investing. Passive investment – investment into index funds or exchange-traded funds (ETFs), whose portfolios track a pre-specified group of assets – has been largely concentrated in the hands of the ‘Big Three’ asset managers, which include BlackRock, Vanguard and State Street Global Advisors, who between them manage nearly $20 trillion in assets (Thinking Ahead Institute, 2021b). These shifting dynamics have important implications for corporate governance and the ways investors can shape company attention to ESG (environment, social, and governance) and climate concerns.

Some have argued that the presence of large asset managers and index funds offers appealing routes through which to address sustainability and climate issues at companies. As the ‘new permanent universal owners,’ with exposure to markets as a whole and an inability to divest from companies (due to tracking an index), these actors arguably have an interest in protecting the long-term sustainability of companies across all sectors (Fichtner & Heemskerk, 2020). Complemented with the majority voting block that the Big Three hold– as much as 25% of votes at S&P 500 companies– this suggests that they may be highly relevant for climate issues (Bebchuk & Hirst, 2019). Dordi et al. (2022), through a network analysis of global FF shareholders, argue that the Big Three are among the most influential global FF shareholders, based on the emissions associated with their investments and their centrality in the network of FF shareholders. The Big Three asset managers are publicly emphasizing their attention to shareholder engagement and stewardship, through means such as BlackRock CEO Larry Fink’s highly publicized annual letters to shareholders and CEOs, creation of ESG funds, increasing transparency into their stewardship activities, and joining sustainability-related investor engagement initiatives, such as Climate Action 100+ (Strampelli, 2020). One study finds an association between ownership by the Big Three (driven by inclusion of a company in a major index) and a subsequent reduction in the company’s carbon emissions, indicating some effectiveness in their environmental stewardship (Azar et al., 2021).

However, others argue that there are limitations in the lengths to which asset managers should be expected to act in the best interest of the shareholder (or indeed, the climate). Most passive asset managers make their money through fees rather than through investment performance and thus are ultimately motivated to expand their base of assets under management (and thus, their fees), rather than to improve the underlying economic performance of those assets (Braun, 2020; Strampelli, 2020). The cost of shareholder engagement is likely not worth the marginal economic gains that could result from such engagement, an argument backed up by the relatively small ESG staff that the Big Three employ. For example, Strampelli (2020) points out that BlackRock spends just 0.15% of their collected asset management fees on stewardship activities (p. 14), while Griffin (2019) highlights that they employ a stewardship team of just 36 people to research and vote on over a hundred thousand shareholder proposals each year (p.10). Gunningham (2020, p. 11) cites research from the NGO InfluenceMap, which finds that only 2 of the top 15 global asset managers engage ‘strongly and consistently’ with companies on alignment with the Paris Agreement. Their voting record on climate issues is similarly poor; grey literature from NGOs indicates that asset managers maintain a tendency to vote with management, against climate-related shareholder proposals (Reclaim Finance et al., 2022; ShareAction, 2021). There are also potential conflicting interests at play, as many companies’ private pension schemes are potential clients for asset managers, which could influence their engagement with those companies (Braun, 2020; Gunningham, 2020). For active asset managers, incentive structures and performance fees are based on very short-term periods, meaning that the longer term impacts associated with climate are ‘heavily discount[ed], or ignore[d] entirely’ (Gunningham, 2020, p. 7). Despite the structural disincentives for asset managers to engage with companies, investing through the services of large asset managers often results in investors (asset owners) effectively handing over their voting rights to those asset managers. This transfer of investor voice to a small number of large asset managers has been criticized as undemocratic and puts enormous power in the hands of a few for-profit actors, who may be subject to various conflicts of interest (McGaughey, 2021).

Since passive investments track a predefined index, the option for divestment is essentially removed. Potentially for this reason, passive investment has been relatively ignored by investors seeking to incorporate climate-related decision making into their investment. Christophers (2019) argues that ‘indexing effectively represents, one might say, not thinking about fossil fuel risk. To the degree that it entails casting in one’s lot with the market, indexing amounts to an abdication of choice in favor of systematized convention (p. 770).’ Gunningham (2020) contends that if large passive asset managers were to shift the majority of their managed assets into low-carbon indices, the transition to a low-carbon financial system could be greatly accelerated. However, he recognizes the improbability that asset managers would undertake this step without significant ‘social license’ pressure from the public, which might threaten asset managers’ reputation. Zeidan (2022) argues that asset managers will not take steps to become more sustainable without demand and pressure from asset owners. In sum, although asset managers are well-placed to take meaningful climate action, there are significant disincentives for them to do so; stronger intervention from both asset owners and policymakers is likely needed to align their investments with climate goals.

#### 3.3.2. Investment consultants

Investment consultants are important in advising and directing pension funds and other investors on ‘the selection and monitoring of investment managers, the selection of benchmarks against which fund managers’ performance can be judged, investment time horizons and asset allocation analysis’ among other services (Pfeifer & Sullivan, 2008, p. 249). While growing attention to climate from large investment consulting companies has been noted (Sullivan & Pfeifer, 2008), their influence on pension fund climate strategies has been relatively ignored by the literature. Knight and Dixon’s (2011) survey of some of the largest investment consultants finds that while some investment consultants were equipped to provide clients with advice on integrating ESG considerations into their investment strategies, there remain many gaps in consultant knowledge and expertise on ESG issues, and many barriers for integrating them. These gaps include cases in which consultants address ESG concerns only when clients specifically request them to (rather than proactively), reliance on assessing performance through benchmarking and tracking error limits, and challenges overcoming the short-termism of fund managers. Yoshino et al. (2021) examine how three major consulting firms understand and measure the SDGs, finding that their differing interpretations have the capacity to distort portfolio allocations. Finally, Taylor (2022, p. 8) interviews actuaries and consultants in the UK, finding that ‘those who understand the facts of climate change and elements of the relationship between climate change and financial risk are in a minority’ and are often constrained by the priorities of asset managers and trustees, despite the position of influence that consultants hold. Taylor (2022) argues that the reliance of the profession on traditional financial tools fails to sufficiently incorporate and address climate risks and calls for greater integration of scenarios which incorporate climate science in their investment advice. Despite the influential role that investment consultants can play in shaping investor strategies, the limited recent research on their attention to climate change indicates an important gap in knowledge.

### 3.4. Investment in the financial sector

While pension funds and other investors frequently hire asset managers to manage their portfolios or invest in asset managers’ funds, they may also invest in asset management companies themselves. For example, a pension fund might invest in a standard passive fund managed by BlackRock, but might also invest in shares in BlackRock, which is itself a publicly listed company. Investment in financial companies, who themselves invest in FF companies, may result in additional indirect exposure to FF companies for investors. Battiston et al.’s (2017) analysis of the exposure of the financial sector to climate risks finds that while investors in the EU and US have a relatively low direct equity exposure to FF (4-13% of their equity holdings), they have significant investments in ‘climate-policy-relevant’ sectors (36-48%) and in the financial sector (13-25%). The interconnectedness of the financial sector amplifies the potential climate risks for all investors. Indirect investment in FF companies (through investment in banks or asset managers who invest in FF) presents a potential additional, though under-addressed, avenue for investors to influence the FF sector. Investment banks, for example, are hugely important to the FF sector, providing approximately 64% of their fundraising through syndicated loans, in addition to underwriting the bonds and equities issued (Cojoianu et al., 2021, p. 2). The 60 largest banks (both investment and commercial banks) have provided the FF industry with $4.6 trillion since the adoption of the Paris Agreement (RAN, 2022, p. 3). Urban and Wójcik (2019, p. 18) see a role for institutional investors to influence not only FF companies, but also the banks financing them: ‘we think there is a real opportunity for large institutional investors to actively engage banks servicing dirty companies and push them to improve their ESG standards in underwriting.’ Institutional investors might amplify the impacts of their shareholder engagement by engaging not only with FF companies, but also with their financiers.

### 3.5. Indirect actors

Though underexplored, the reviewed literature indicates the relevance of indirect financial actors for shaping investor strategies and their capacity for acting on climate change. We define indirect financial actors as those who function as service providers to investors (here, namely, index providers and investment advisory firms) or facilitators of investment (e.g. stock exchanges).

#### 3.5.1. Index providers

With the growth in passive investing (see 3.3.1), indices and index providers will play an increasingly influential role. Indices can be created on an ad hoc basis by investors themselves, which has often been done to create options for ‘socially responsible’ funds (Strampelli, 2020). However, many large funds and ETFs track standard third-party indices, such as the S&P 500 or Russell 3000. The index providers who create and define these indices thus ‘act as genuine gatekeepers that are capable of influencing the actions of listed companies in a manner that is in some respects more far reaching than institutional investors’ (Strampelli, 2020, p. 17). The influence of index providers is highly consolidated, with three main providers, MSCI, S&P Dow Jones Indices, and FTSE Russell controlling 80% of the market share (ibid). Index providers have significant power to direct investment flows into sustainable or green options, through how they choose to measure, weigh, and promote sustainability related company criteria. Their choices impact the availability of financing not only for companies, but also for countries, based on how emerging markets are assessed and included or excluded from indices (Van de Putte et al., 2020). Due to demand for ESG options, index providers are increasingly creating various sustainability-oriented indices, such as the MSCI Low Carbon Leaders Index (Gunningham, 2020). Strampelli (2020) points out that major asset managers like BlackRock have signalled their intention to collaborate with index providers to create more sustainable options, but is pessimistic about the likelihood that index providers would use their gatekeeping role to exclude companies from their most popular indices.

#### 3.5.2. Stock exchanges

Stock exchanges function as the marketplaces on which stocks, bonds, and other financial products can be traded. They set requirements for companies to be listed on their exchange, which allows them to shape aspects of the market. While their role with respect to climate has been underexplored, several papers point out that stock exchanges, in addition to securities regulators (e.g. the Securities and Exchange Commission (SEC) in the US) could theoretically require companies to comply with more stringent climate-related requirements in order to be listed on the exchange (e.g. mandatory disclosure of emissions or climate risks) (Bebbington et al., 2020; Puri, 2019). There is some evidence of investors engaging with exchanges, such as a 2014 proposal from the investor initiative Ceres, which suggests that exchanges adopt a range of listing standards for ESG disclosures (Cleary, 2015).

#### 3.5.3. Proxy advisory firms

As most institutional investors invest in thousands of companies, they will submit thousands of votes every year, on shareholder resolutions and other topics, including board or director confirmations, and financial statement and auditor approval votes. Given the vast number of votes investors are expected to submit, and the limited resources generally available to their corporate governance teams (see also 3.3.1), many utilise the services of investment or proxy advisory firms for their ‘expert independent insights’ (Hoepner et al., 2022, p. 4). The market for proxy advisory firms is dominated by just two companies, Institutional Shareholder Services (ISS) and Glass Lewis, who together control over 90% of the proxy advisory market (Hoepner et al., 2022, p. 7). These two companies thus occupy a position of significant power, as many institutional investors not only use their services, but also vote automatically in line with their recommendations. While the influence of proxy advisory firms on corporate governance has been examined (e.g. Larcker et al., 2015), the extent to which their recommendations consider climate and their role in supporting or hindering climate action from companies has been relatively unstudied. Hoepner et al. (2022) study how proxy advisory firms consider climate in their recommendations for voting on proposals at 78 emissions-intensive companies. They find that while ISS and Glass Lewis use comparisons to industry peers or consideration of material as factors influencing their recommendations for climate-specific proposals, their recommendations for votes on financial accounting do not consider the alignment of those financial plans with climate goals.

The very limited literature on these indirect actors presents similar patterns and gaps to the literature on asset managers and consultants. Investor allocation of assets and their engagement with the companies they invest in is often shaped by, and in some cases outsourced to, a consolidated number of for-profit actors with potentially differing incentives and accountabilities than those of the institutional investors they represent. This may raise challenges for relying on investor action to address climate concerns and for directing finance towards avenues which accelerate the energy transition and encourage inclusive development. These concerns merit further research into the governance of indirect financial actors, through public policy as well as through the powerful financial actors who engage their services.

### 3.6. Litigation

Although the number of climate change-related court cases has been growing rapidly since the early 2000s, litigation by investors or targeting investors, or litigation based on claims of misleading investors or breaching fiduciary duties, are all very recent trends (Setzer & Higham, 2022). Several reviewed articles raise the issue of litigation, both as a potential tool and risk for investors. Bruno (2019) cites two US court cases (as yet unresolved) in which FF companies, or their boards of directors, were charged with breaching their fiduciary duties to shareholders. Although such cases could influence FF companies, Bruno (2019) argues that the feasibility of winning them is contested, especially in the US. However, court cases initiated by shareholders could serve to generate negative publicity for high carbon companies and contribute pressure threatening FF companies’ social license to operate (Gunningham, 2020).

The threat of litigation from their own beneficiaries could also motivate institutional investors to increase the extent to which they integrate climate risks and action into their decision making (Pearce, 2021). Pension funds and many other investors are governed by fiduciary duty laws, which require funds to act in the best interest of their beneficiaries and invest prudently and diligently. Traditional interpretations require investors to consider financial returns above all, which in practice often means following what the majority of investors do, and upholding the status quo (Woods, 2011). While there have been increasing calls to integrate ESG considerations into understandings of fiduciary duty, these still require ESG issues to be financially materially relevant; investors would not be allowed to make investments purely on an ethical or sustainable basis, without consideration of the financial risks or opportunities (Sørensen & Pfeifer, 2011; Strakodonskaya, 2021). The Australian case *McVeigh* v. *Retail Employees Superannuation Trust*, in which a pension holder sued their pension fund for failing to manage climate risk, provides an example of updated interpretations of fiduciary duty. In a settlement out of court, the fund agreed to comply with disclosure and ‘due diligence climate-related duties in accordance with the recommendations of the [TCFD],’ though these commitments are not legally binding (Colombo, 2022, p. 175). Through such settlements, courts may ‘articulate a duty, rather than simply permit pension funds to consider climate-related financial risk’ (Colombo, 2022, p. 184). Reconsideration and redefinition of fiduciary duties will be an important step in enabling and encouraging investors to take more ambitious climate action, especially if it implies costs in the short- to medium-term.

### 3.7. Green investment

A major role for investors in driving the energy transition is in providing the finance needed to scale up renewable energies and green infrastructure, as well as the greening of industry. While other reviews have conducted bibliometric analyses of green finance research (Zhang et al., 2019) and examined the differences in global adoption of green finance (Ozili, 2022), we focus here on how the literature addresses green investment as a tool of climate mitigation and a contributor to phasing out FF. The IEA estimates that approximately $4 trillion in investment will be needed annually by 2030 (current levels are approximately $1 trillion annually), about 70% of which they estimate will need to come from private finance (IEA, 2021). However, the current percentage of institutional investors’ assets that are allocated to ‘green infrastructure’ remains low (estimated to be below 1% for OECD pension funds (Studart & Gallagher, 2018)). Despite the seeming potential to match the vast resources controlled by institutional investors with green financing needs, authors cite various barriers preventing greater investment in renewable energy or green projects. These barriers include rate and reliability of returns on investment (Côté & Salm, 2022); the small size, complexity, relative illiquidity of renewable energy projects, as well as the differing needs for financing at various project stages (Ameli et al., 2020; Hebb, 2018; Polzin & Sanders, 2020; Rezec & Scholtens, 2017; Van de Putte et al., 2020); uncertain risk levels, uncertain regulatory environments, and limited performance data (Ameli et al., 2020; Hebb, 2018; Ozili, 2022); lack of capacity for investors to research smaller projects (Ameli et al., 2020); and market structures, such as index composition which direct investments to certain markets and sectors to the exclusion of others (Van de Putte et al., 2020). These papers agree that a rapid scaling up of investment in renewables and other green sectors will not happen on its own – government intervention will be needed. Suggested government interventions include: taking steps to de-risk renewables projects (Gitonga & Ali, 2018), increasing public-private partnerships, green bond issuance, aggregation of renewable projects to make them more investable for institutional investors, use of feed in tariffs, creation of new asset classes (Mielke, 2019), and competitive pricing for green bonds (Sangiorgi & Schopohl, 2021), among others. Cullen and Mähönen (2019) propose increasing green investments through measures to discourage financing of climate-damaging projects, namely: increasing the level and stringency of climate stress-testing for banks; raising capital requirements for high-carbon investments; and targeting green sectors through quantitative easing and other central bank initiatives.

While a thorough engagement with the relative strengths and weaknesses of each of these approaches is beyond the scope of this paper, and has been undertaken elsewhere (see e.g. Polzin et al., 2019; Rempel & Gupta, 2021), two conclusions are relevant. First, there is a need to adopt strategies to address ‘dirty’ investments and ‘green’ investments simultaneously, but separately. Assuming that the climate strategies adopted by investors –especially measuring climate risk, or increasing transparency and reporting– will effectively direct finance away from polluting industries and into greener ones is misguided (Ameli et al., 2020; Gunningham, 2020). Second, most suggested policies to encourage green finance rely on improving the ‘bankability’ of green investments. In most forms, this requires governments and public institutions to take on the risks associated with new projects, in order to ensure a sufficient level of return to investors (Gabor, 2021). Given the uneven distribution of wealth globally, much of which is controlled by institutional investors in the Global North, policies that encourage developing country governments to take on the costs and risks associated with attracting finance raise significant concerns over equity and fair distribution of financial flows.

### 3.8. Discussion

**Figure 2.**
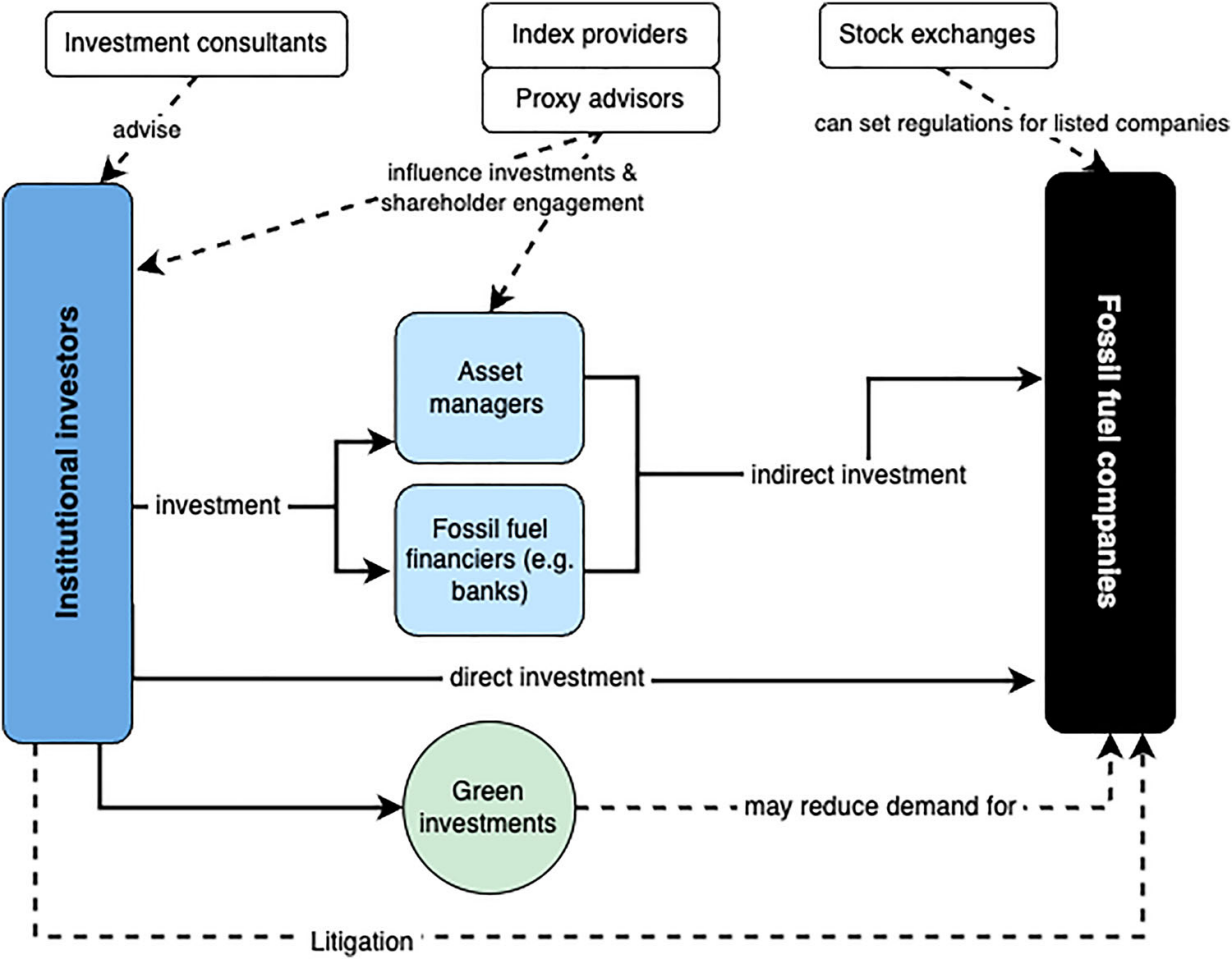
Key actors and strategies, draws on (Braun, 2020). Solid lines represent financial investments; dotted lines represent non-investment ties.

This review reveals that the scope of potential institutional investor climate action is much broader than is often portrayed when focusing on investors’ role as shareholders of high-carbon companies. In this review, we identify a range of potential strategies that institutional investors might pursue to amplify their climate action. Although they are presented separately in this review, these strategies could certainly be adopted in coordination with each other. An investor’s governance strategy could include engagement with FF companies, engagement with FF financiers, incorporating specific criteria on FF into their mandates for asset managers and consultants, lobbying or selectively choosing how they use the services of indirect financial actors, and even litigation, although each will require some level of investment of time and resources. Divestment may also be included in governance strategies, as a ‘stick’ used in the cases of unsuccessful engagement, or as a selective tool, applied to the worst offenders in a sector (Edmans et al., 2022). Green investment could take place alongside the other strategies, as well as post-divestment, by reinvesting divested funds into green alternatives. There are also certain interdependencies between strategies. For example, adjusting mandates for asset managers, or implementing changes at proxy advisors or index providers could result in changes to engagement strategies and voting practices, potential divestments, and changes in levels of green investment. Figure 2 illustrates some of the connections between institutional investors, key actors and strategies, and FF companies identified in this paper, while Table 2 summarizes key strengths and weaknesses of each strategy. While we have centred institutional investors, this review also illustrates that investors, and more specifically major asset owners like pension funds, are but one part of a broader financial system that contributes in varying ways to uphold the FF sector. While activists have in recent years begun to draw attention to the role of actors like asset managers and banks in supporting the FF industry (see e.g. BlackRock’s Big Problem, n.d.; RAN, 2022), these actors have received relatively little attention from academic research.

**Table 2.** Strengths and weaknesses of identified strategies for phasing out FF

Strategy	Strengths	Weaknesses
Divestment	May have reputational consequences for FF firms May financially benefit investors Useful as a tool used selectively in coordination with other strategies	Unlikely to restrict FF companies’ access to finance May result in reduced transparency of FF shareholders May result in inequitable distribution of stranded assets
Shareholder engagement with FF companies	Investors have continuous oversight over FF companies Engagement can take various forms, allowing investors to escalate their demands through the engagement process	Investors may pursue unambitious engagement strategies (e.g. requests for disclosure) Unambitious investor engagements risk delaying more ambitious climate action by appearing to take action
Hiring Practices	Investors could multiply the effectiveness of their engagement strategies by incorporating climate-related criteria into mandates for asset managers and consultants	Consolidation of the asset management and consultancy sectors to a few large actors may increase difficulty of influencing them The incentives of asset managers and consultants may not align with investor incentives
Shareholder engagement with FF financiers	Targeting FF financiers (e.g. banks) through engagement could impact FF companies’ access to finance	Investors may pursue unambitious engagement strategies
Engaging indirect actors	Many indirect actors, through their influential position, could have a disproportionate impact if they adopted climate-related policies	Little is known about strategies and opportunities to influence indirect actors; this strategy may hold uncertainties and require further research
Litigation	Opportunity to hold FF companies to a binding result	Likelihood of winning lawsuits could vary significantly based on region and type of case
Green investment	Provides much-needed finance for renewable energy and other green sectors	Green investment alone will likely not automatically reduce investment in FF Must be pursued in conjunction with strategies to phase out FF Available investment avenues may not align with the needs of renewable projects

By engaging with the literature through an inclusive lens, our review also identifies potential implications and limitations of investors strategies for contributing to an inclusive fossil fuel phase-out. A consistent and cross-cutting limitation is the prioritization of financial returns. The focus specifically on short-term financial returns has been a barrier to more ambitious forms of shareholder engagement on climate issues, as well as a barrier to allocating greater levels of finance towards sustainable, green investments. While not an unexpected finding, it underscores the need for academics and policymakers to seriously engage with the reality that trade-offs will likely have to be made between social and environmental outcomes and economic ones. It also indicates that voluntary investor efforts alone, through mechanisms like engagement, disclosure, and ESG investing, will not overcome the prioritization of financial returns. Though this is a requirement of fiduciary duty laws, as they are currently understood, it limits the potential or likelihood that investors will take action aligned with a rapid FF phase-out, or inclusive outcomes. This suggests that policy (rather than initiative from the financial sector alone) is needed to address the thorny issue of redirecting the incentive structures that guide investors away from prioritizing near-term profit maximization at the expense of preventing climate catastrophe and protecting long-term social and ecological wellbeing.

With respect to social inclusiveness, there remain many barriers to connecting the vast amounts of institutional investor capital available with the sectors, projects, and countries where it is most needed. Allocation of institutional investor finance is determined not only by the many factors affecting the ‘bankability’ of certain projects, but also greatly influenced by the decisions made by index providers and asset managers. With respect to ecological inclusiveness, focusing specifically on contributions to climate change mitigation through phasing out FF, the literature indicates that investor strategies have thus far been inadequate, and generally disproportionate to the scale of action needed to mitigate the worst impacts of climate change. Beyond advocating generally for the need for more radical action from institutional and financial actors, we also argue that a more explicit focus on FFs is needed to effectively work towards meeting climate goals. We note that much of the relevant literature and action from investors relies on proxy measures (such as emissions) alone, rather than focusing concretely on ending FF exploration and expansion and rapidly cutting production. While emissions measurements have their purpose, their use in creative accounting which obscures FF expansion should be challenged (Green & Kuch, 2022). Investors could take this on by focusing engagements on metrics such as ceasing financing for new FF projects, rather than setting emissions targets only. We also see an opportunity for academics to contribute to shifting this narrative by better contextualizing research which relies on emissions metrics. While many studies assess the impact of investor actions on emissions levels or intensity, they rarely contextualize results against both actual emissions cuts (compared to e.g. reduced portfolio emissions) and against the levels of emissions reductions that are needed to meet climate goals. Finally, with respect to relational inclusiveness, this review identified a trend of consolidation of decision-making power into a small number of (often US-based) for-profit actors. This trend may limit the inclusiveness of access to and control over finance’s involvement in both fossil and green sectors. We also note a lack of attention to how the decisions taken by institutional investors based overwhelmingly in the Global North may have ramifications for climate and development outcomes in the Global South. Given the current centrality of institutional investors in plans for financing global climate and development goals, this is an important area for further research.

## 4. Conclusion

This review has identified and explored seven strategies available to institutional investors for influencing the FF sector: (a) divestment; (b) shareholder engagement with FF companies; (c) engaging with their asset managers and investment consultants; (d) engaging with the FF financiers they invest in; (e) engaging with indirect financial actors; (f) litigation; and (g) increasing green investments. While each strategy could potentially contribute to phasing out FFs, their effectiveness is often limited by the mandates and/or incentive structures which encourage investors to prioritize short-term profits. While we see much scope for more ambitious investor action, we also argue that without regulation or interventions to require investors to take a longer-term perspective, voluntary investor action alone will likely be insufficient to meet climate goals. Thus, while we focus in this review on the role for institutional investors, these strategies also identify important areas for further attention and research from academics, activists, and policy makers. More research into how policies can incentivise investor engagement with the strategies identified, while discouraging trade-offs in favour of short-term financial returns, will be needed. The role of the various and understudied financial actors discussed in this review, as well as the implications of actions taken by the financial sector for social inclusiveness and development, are also important areas for further attention from scholars.

## Supplementary Material

Supplemental Material

## Footnotes

1 For reference, global GDP in 2023 is estimated at $106 trillion (IMF, 2023), while global net worth (all real and financial assets) at the end of 2021 was $610 trillion (Mischke et al., 2022).

2 Institutional investors are legal entities that invest on behalf of their clients or beneficiaries and can take a variety of forms, from hedge funds to endowment funds. Although we focus on pension funds and asset managers as particularly relevant investors, the strategies we identify are sufficiently broad as to be relevant for other institutional investors.

3 Although we recognise that different investor types will have different motivations and strategies, exploring these distinctions is not the purpose of this review. Thus, we follow the cues of the literature cited, referring generally to institutional investors, and clarifying investor type when specified by the cited authors.

4
https://www.rayyan.ai/

5 Including: LNG, lignate, decarb*, carbon, net-zero.

6 Citation searching identifies relevant papers by checking the reference lists of included papers; pearl-growing uses key papers to identify further relevant papers, through reference links or suggested or similar articles presented by online databases.

7 We use the terms ‘Global North’ and ‘Global South’ to refer in general to the distinction between high-income and low- and middle-income countries respectively, while acknowledging the relevance of geographies and histories of imperialism in shaping those distinctions. While we recognise that any such division is oversimplified and obscures national and regional inequalities, as well as other nuances, we believe that especially in the context of the financial sector and the climate crisis, examining inequality through the Global North-Global South frame is critical (Sultana, 2022).
