# Supplementary material for: Beyond divest vs. engage: a review of the role of institutional investors in an inclusive fossil fuel phase-out
Source: Clim Policy. 2023 Oct 12;24(3):314–31. doi: 10.1080/14693062.2023.2261900 (PMC10962715; doi:10.1080/14693062.2023.2261900)
Supplement: Supplemental Material [file TCPO_A_2261900_SM9632.docx]

Beyond divest vs. engage: a review of the role of institutional investors in an inclusive fossil fuel phase-out

Clara McDonnell^^[[1]](#footnote-1)^^, Joyeeta Gupta^^[[2]](#footnote-2)^^

# Appendix

## Literature review search terms

| Query | Search Terms | Search Results | Articles Selected |
| --- | --- | --- | --- |
| 1 | (pension OR “institutional investor” OR “asset manager”) AND (climate OR “fossil fuel” OR “oil” OR “coal” OR “gas”) | 804 | 96 |
| 2 | divestment AND ("fossil fuel" OR oil OR gas OR coal OR climate) | 259 | 14 |
| 3 | ((shareholder AND (engag* OR proposal OR resolution OR advoc*)) AND ("fossil fuel" OR oil OR gas OR coal OR climate)) | 121 | 6 |
| 4 | investor AND consultant AND ("fossil fuel" OR oil OR gas OR coal OR climate)) | 36 | 1 |
| 5 | “stock exchange” AND ("fossil fuel" OR climate)) | 126 | 0 |
| 6 | “proxy advisory” AND ("fossil fuel" OR climate)) | 0 | 0 |
| 7 | “index provider” AND (“fossil fuel” OR oil OR gas OR coal OR climate) | 0 | 0 |
| 8 | litigation AND investor AND ("fossil fuel" OR oil OR gas OR coal OR climate) | 36 | 3 |
| 9 | "green investment" OR "sustainable investment") AND ("fossil fuel" OR oil OR gas OR coal OR climate) AND investor | 83 | 8 |
|  | Citation searching, pearl-growing | n/a | 19 |
|  | **Total** | **1382** | **147** |

## Reviewed articles

| **Number** | **Publication Year** | **Author** | **Title** | **Publication** |
| --- | --- | --- | --- | --- |
| 1 | 2021 | Acar, Ece; Tunca Çalıyurt, Kıymet; Zengin-Karaibrahimoglu, Yasemin | Does ownership type affect environmental disclosure? | International Journal of Climate Change Strategies and Management |
| 2 | 2014 | Ahmed, Sarwar Uddin; Islam, Zahidul; Mahtab, Hanif; Hasan, Ikramul | Institutional Investment and Corporate Social Performance: Linkage towards Sustainable Development | Corporate Social Responsibility and Environmental Management |
| 3 | 2022 | Al Ayoubi, Khalil; Enjolras, Geoffroy | Does disinvestment from fossil fuels reduce the financial performance of responsible sovereign wealth funds? | Journal of Multinational Financial Management |
| 4 | 2020 | Ameli, Nadia; Drummond, Paul; Bisaro, Alexander; Grubb, Michael; Chenet, Hugues | Climate finance and disclosure for institutional investors: why transparency is not enough | Climatic Change |
| 5 | 2022 | Arslan, Hafiz Muhammad; Chengang, Ye; Bilal; Siddique, Muhammad; Yahya, Yusra | Influence of Senior Executives Characteristics on Corporate Environmental Disclosures: A Bibliometric Analysis | Journal of Risk and Financial Management |
| 6 | 2017 | Ayling, Julie; Gunningham, Neil | Non-state governance and climate policy: the fossil fuel divestment movement | Climate Policy |
| 7 | 2021 | Azar, José; Duro, Miguel; Kadach, Igor; Ormazabal, Gaizka | The Big Three and corporate carbon emissions around the world | Journal of Financial Economics |
| 8 | 2018 | Banda, Maria L. | The bottom-up alternative: The mitigation potential of private climate governance after the paris agreement | Harv. Envtl. L. Rev. |
| 9 | 2021 | Bassen, Alexander; Kaspereit, Thomas; Buchholz, Daniel | The Capital Market Impact of Blackrock’s Thermal Coal Divestment Announcement | Finance Research Letters |
| 10 | 2017 | Battiston, Stefano; Mandel, Antoine; Monasterolo, Irene; Schütze, Franziska; Visentin, Gabriele | A climate stress-test of the financial system | Nature Climate Change |
| 11 | 2018 | Bauer, N.; McGlade, C.; Hilaire, J.; Ekins, P. | Divestment prevails over the green paradox when anticipating strong future climate policies | Nature Climate Change |
| 12 | 2019 | Bebchuk, Lucian; Hirst, Scott | The Specter of the Giant Three | Boston University Law Review |
| 13 | 2022 | Benlemlih, Mohammed; Arif, Muhammad; Nadeem, Muhammad | Institutional Ownership and Greenhouse Gas Emissions: A Comparative Study of the UK and the USA | British Journal of Management |
| 14 | 2018 | Bergman, N. | Impacts of the fossil fuel divestment movement: Effects on finance, policy and public discourse | Sustainability (Switzerland) |
| 15 | 2021 | Bernardini, Enrico; Di Giampaolo, Johnny; Faiella, Ivan; Poli, Riccardo | The impact of carbon risk on stock returns: evidence from the European electric utilities | Journal of Sustainable Finance & Investment |
| 16 | 2021 | Bhopal, Anand | The Norwegian Oil Fund in a Warming World: What are the Interests of Future Generations? | Ethics, Policy & Environment |
| 17 | 2019 | Boermans, Martijn A.; Galema, Rients | Are pension funds actively decarbonizing their portfolios? | Ecological Economics |
| 18 | 2021 | Bolton, Patrick; Kacperczyk, Marcin T. | Do investors care about carbon risk? | Journal of Financial Economics |
| 19 | 2020 | Bolton, Patrick; Kacperczyk, Marcin T. | Signaling through Carbon Disclosure | SSRN Electronic Journal |
| 20 | 2021 | Bolton, Patrick; Kacperczyk, Marcin T. | Firm Commitments | SSRN Electronic Journal |
| 21 | 2019 | Brauch, Martin Dietrich; Touchette, Yanick; Cosbey, Aaron; Gerasimchuk, Ivetta; Sanchez, Lourdes; Bernasconi-Osterwalder, Nathalie; Garcia, Maria Bisila Torao; Potaskaevi, Temur; Petrofsky, Erica | Treaty on Sustainable Investment for Climate Change Mitigation and Adaptation: Aligning International Investment Law with the Urgent Need for Climate Change Action | Journal of International Arbitration |
| 22 | 2020 | Braun, Benjamin | Asset Manager Capitalism as a Corporate Governance Regime | The American Political Economy: Politics, Markets, and Power |
| 23 | 2019 | Braungardt, Sibylle; van den Bergh, Jeroen; Dunlop, Tessa | Fossil fuel divestment and climate change: Reviewing contested arguments | Energy Research & Social Science |
| 24 | 2019 | Bruno, Sabrina | Climate Corporate Governance: Europe vs. USA? | European Company and Financial Law Review |
| 25 | 2013 | Calza, Francesco; Profumo, Giorgia; Tutore, Ilaria | Does corporate ownership structure affect firms’ environmental performance? Evidence in the European energy industry | International Journal of Globalisation and Small Business |
| 26 | 2022 | Canal Vieira, Leticia; Longo, Mariolina; Mura, Matteo | Will the regime ever break? Assessing socio-political and economic pressures to climate action and European oil majors’ response (2005-2019) | Climate Policy |
| 27 | 2020 | Chithambo, Lyton; Tingbani, Ishmael; Agyapong, Godfred Afrifa; Gyapong, Ernest; Damoah, Isaac Sakyi | Corporate voluntary greenhouse gas reporting: Stakeholder pressure and the mediating role of the chief executive officer | Business Strategy and the Environment |
| 28 | 2019 | Christophers, Brett | Environmental beta or how institutional investors think about climate change and fossil fuel risk | Annals of the American Association of Geographers |
| 29 | 2005 | Clark, Gordon L.; Hebb, Tessa | Why should they care? The role of institutional investors in the market for corporate global responsibility | Environment and planning A |
| 30 | 2004 | Clark, Gordon; Hebb, Tessa | Pension Fund Corporate Engagement: The Fifth Stage of Capitalism | Relations industrielles / Industrial Relations |
| 31 | 2021 | Cojoianu, T.F.; Hoepner, A.G.F.; Schneider, F.I.; Urban, M.; Vu, A.; Wójcik, D. | The city never sleeps: but when will investment banks wake up to the climate crisis? | Regional Studies |
| 32 | 2021 | Cojoianu, Theodor F.; Ascui, Francisco; Clark, Gordon L.; Hoepner, Andreas GF; Wójcik, Dariusz | Does the fossil fuel divestment movement impact new oil and gas fundraising? | Journal of Economic Geography |
| 33 | 2022 | Colombo, Esmeralda | From Bushfires to Misfires: Climate-related Financial Risk after McVeigh v. Retail Employees Superannuation Trust | Transnational Environmental Law |
| 34 | 2015 | Combet, Emmanuel; Hourcade, Jean Charles | Carbon tax, pensions and public deficits: The hidden costs of the compartmentalization of expertise | 3ème conférence annuelle de la GGKP |
| 35 | 2022 | Côté, Elizabeth; Salm, Sarah | Risk-adjusted preferences of utility companies and institutional investors for battery storage and green hydrogen investment | Energy Policy |
| 36 | 2012 | Cotter, Julie; Najah, Muftah M | Institutional investor influence on global climate change disclosure practices | Australian Journal of Management |
| 37 | 2013 | Cotter, Julie; Najah, Muftah M. | Corporate climate change disclosure practices and regulation: The influence of institutional investors | Institutional Investors’ Power to Change Corporate Behavior: International Perspectives |
| 38 | 2019 | Cullen, Jay T.; Mähönen, Jukka | Taming unsustainable finance: The perils of modern risk management | Cambridge Handbook of Corporate Law, Corporate Governance, and Sustainability |
| 39 | 2020 | Curran, Giorel | Divestment, energy incumbency and the global political economy of energy transition: the case of Adani’s Carmichael mine in Australia | Climate Policy |
| 40 | 2021 | Curtis, Quinn; Fisch, Jill E.; Robertson, Adriana | Do ESG Mutual Funds Deliver on Their Promises? | SSRN Electronic Journal |
| 41 | 2021 | D’Amato, Valeria; D’Ecclesia, Rita; Levantesi, Susanna | ESG score prediction through random forest algorithm | Computational Management Science |
| 42 | 2020 | Davies, Emily | Recommendations for Effectively Resolving Climate Change Disputes against Investors | Carbon & Climate Law Review (CCLR) |
| 43 | 2022 | Díaz-Peña, Luz del Carmen; Castillo Delgadillo, Victor Manuel; Mario Iván, Contreras-Valdez | Financial firm’s performance: a comparative analysis based on ESG metrics and net zero legislation | Journal of Sustainable Finance & Investment |
| 44 | 2020 | Diringer, E.; Perciasepe, B. | The climate awakening of global capital | Bulletin of the Atomic Scientists |
| 45 | 2019 | Dordi, Truzaar; Weber, Olaf | The Impact of Divestment Announcements on the Share Price of Fossil Fuel Stocks | Sustainability |
| 46 | 2021 | Drollette, Dan | Interview: CalPERS’ Anne Simpson on the climate change power of investment managers | Bulletin of the Atomic Scientists |
| 47 | 2022 | Edmans, Alex; Levit, Doron; Schneemeier, Jan | Socially Responsible Divestment | SSRN Electronic Journal |
| 48 | 2022 | Egli, Florian; Schärer, David; Steffen, Bjarne | Determinants of fossil fuel divestment in European pension funds | Ecological Economics |
| 49 | 2021 | El Ouadghiri, Imane; Guesmi, Khaled; Peillex, Jonathan; Ziegler, Andreas | Public Attention to Environmental Issues and Stock Market Returns | Ecological Economics |
| 50 | 2022 | Fahmy, Hany | The rise in investors’ awareness of climate risks after the Paris Agreement and the clean energy-oil-technology prices nexus | Energy Economics |
| 51 | 2022 | Fan, John Hua; Omura, Akihiro; Roca, Eduardo | An industry-guided review of responsible investing: Bridging the divide between academia and industry | Journal of Cleaner Production |
| 52 | 2022 | Faria, João Ricardo; Tindall, Greg; Terjesen, Siri | The Green Tobin's q: theory and evidence | Energy Economics |
| 53 | 2020 | Fichtner, Jan; Heemskerk, Eelke M. | The New Permanent Universal Owners: Index funds, patient capital, and the distinction between feeble and forceful stewardship | Economy and Society |
| 54 | 2021 | Flammer, Caroline; Toffel, Michael W.; Viswanathan, Kala | Shareholder activism and firms' voluntary disclosure of climate change risks | Strategic Management Journal |
| 55 | 2021 | Foerster, Anita; Sheehan, Kym; Parris, Daniel | Investing for a safe climate? | The University of New South Wales Law Journal |
| 56 | 2021 | Gabor, Daniela | The Wall Street Consensus | Development and Change |
| 57 | 2018 | Gitonga, Stephen; Ali, Walid | De-risking low carbon investments in the GCC | The Economics of Renewable Energy in the Gulf |
| 58 | 2018 | Glomsrød, Solveig; Wei, Taoyuan | Business as unusual: The implications of fossil divestment and green bonds for financial flows, economic growth and energy market | Energy for Sustainable Development |
| 59 | 2022 | Golland, Ami; Galaz, Victor; Engstrom, Gustav; Fichtner, Jan | Proxy Voting for the Earth System: Institutional Shareholder Governance of Global Tipping Elements |  |
| 60 | 2013 | Gond, Jean-Pascal; Piani, Valeria | Organizing the collective action of institutional investors: Three case studies from the principles for responsible investment initiative | Institutional Investors’ Power to Change Corporate Behavior: International Perspectives |
| 61 | 2022 | Gözlügöl, Alperen A | The clash of ‘E’ and ‘S’ of ESG: just transition on the path to net zero and the implications for sustainable corporate governance and finance | The Journal of World Energy Law & Business |
| 62 | 2021 | Grabinska, Barbara; Kedzior, Marcin; Kedzior, Dorota; Grabinski, Konrad | The Impact of Corporate Governance on the Capital Structure of Companies from the Energy Industry. The Case of Poland | Energies |
| 63 | 2022 | Greenwood, Noelle; Warren, Peter | Climate risk disclosure and climate risk management in UK asset managers | International Journal of Climate Change Strategies and Management |
| 64 | 2021 | Griffin, Paul A.; David, H. Lont; Pomare, Carol | The curious case of Canadian corporate emissions valuation | The British Accounting Review |
| 65 | 2020 | Gunningham, Neil | A Quiet Revolution: Central Banks, Financial Regulators, and Climate Finance | Sustainability |
| 66 | 2022 | Guo, Xiaozhu; Liang, Chao; Umar, Muhammad; Mirza, Nawazish | The impact of fossil fuel divestments and energy transitions on mutual funds performance | Technological Forecasting and Social Change |
| 67 | 2020 | Gupta, Joyeeta; Rempel, Arthur; Verrest, Hebe | Access and allocation: the role of large shareholders and investors in leaving fossil fuels underground | International Environmental Agreements: Politics, Law and Economics |
| 68 | 2011 | Haigh, Matthew | Climate policy and financial institutions | Climate Policy |
| 69 | 2019 | Halcoussis, D.; Lowenberg, A.D. | The effects of the fossil fuel divestment campaign on stock returns | North American Journal of Economics and Finance |
| 70 | 2020 | Hansen, T.; Pollin, R. | Economics and climate justice activism: assessing the financial impact of the fossil fuel divestment movement | Review of Social Economy |
| 71 | 2015 | Haque, Shamima; Islam, Muhammad Azizul | Stakeholder pressures on corporate climate change-related accountability and disclosures: Australian evidence | Business and Politics |
| 72 | 2011 | Harmes, Adam | The Limits of Carbon Disclosure: Theorizing the Business Case for Investor Environmentalism | Global Environmental Politics |
| 73 | 2017 | Harnett, Elizabeth S. | Social and asocial learning about climate change among institutional investors: lessons for stranded assets | Journal of Sustainable Finance & Investment |
| 74 | 2018 | Hebb, T. | Investing in sustainable infrastructure | Challenges in Managing Sustainable Business: Reporting, Taxation, Ethics and Governance |
| 75 | 2020 | Hestres, L.E.; Hopke, J.E. | Fossil fuel divestment: theories of change, goals, and strategies of a growing climate movement | Environmental Politics |
| 76 | 2022 | Hoepner, Andreas; Mc Kenna, Cormac; McQuade, Matthew; Schneider, Fabiola | Are Proxy Advisory Firms Ignoring Quality of Accounting Failings at Carbon-Intensive Companies? |  |
| 77 | 2013 | Horne, J. Paul | Climate change and economic growth enigma: An investment suggestion from Wall Street | Environmental Innovation and Societal Transitions |
| 78 | 2019 | Hunt, C.; Weber, O. | Fossil Fuel Divestment Strategies: Financial and Carbon-Related Consequences | Organization and Environment |
| 79 | 2021 | Ilhan, Emirhan; Krueger, Philipp; Sautner, Zacharias; Starks, Laura T. | Climate Risk Disclosure and Institutional Investors | SSRN Electronic Journal |
| 80 | 2022 | Janssen, Artjom; Botzen, Wouter; Dijk, Justin; Duijm, Patty | Overcoming misleading carbon footprints in the financial sector | Climate Policy |
| 81 | 2022 | Jonsdottir, Bjorg; Sigurjonsson, Throstur Olaf; Johannsdottir, Lara; Wendt, Stefan | Barriers to Using ESG Data for Investment Decisions | Sustainability |
| 82 | 2021 | Kelly, Tom Giles | Institutional investors as environmental activists | Journal of Corporate Law Studies |
| 83 | 2021 | Khan, Firdaus | Socially Responsible Investing and Sustainable Indices: A Sustainability Agenda | Indian Journal of Corporate Governance |
| 84 | 2011 | Kim, Eun-Hee; Lyon, Thomas | When Does Institutional Investor Activism Increase Shareholder Value?: The Carbon Disclosure Project | The B.E. Journal of Economic Analysis & Policy |
| 85 | 2019 | Klumpes, P.; Acharyya, M.; Kakar, G.; Sturgess, E. | Climate risk reporting practices by UK insurance companies and pension schemes | British Actuarial Journal |
| 86 | 2011 | Knight, Eric RW; Dixon, Adam D. | The role of investment consultants in transforming pension fund decision-making: the integration of environmental, social and governance considerations into corporate valuation | Corporate governance failures: The role of institutional investors in the global financial crisis |
| 87 | 2021 | Kordsachia, Othar; Focke, Maximilian; Velte, Patrick | Do sustainable institutional investors contribute to firms’ environmental performance? Empirical evidence from Europe | Review of Managerial Science |
| 88 | 2020 | Krueger, Philipp; Sautner, Zacharias; Starks, Laura T | The Importance of Climate Risks for Institutional Investors | The Review of Financial Studies |
| 89 | 2017 | Kruitwagen, Lucas; Madani, Kaveh; Caldecott, Ben; Workman, Mark H. W. | Game theory and corporate governance: conditions for effective stewardship of companies exposed to climate change risks | Journal of Sustainable Finance & Investment |
| 90 | 2021 | Langley, P.; Bridge, G.; Bulkeley, H.; van Veelen, B. | Decarbonizing capital: Investment, divestment and the qualification of carbon assets | Economy and Society |
| 91 | 2018 | Lozano, Rodrigo; Reid, Angus | Socially responsible or reprehensible? Investors, electricity utility companies, and transformative change in Europe | Energy Research & Social Science |
| 92 | 2016 | Ma, Vincent C.; Liu, John S. | Exploring the research fronts and main paths of literature: a case study of shareholder activism research | Scientometrics |
| 93 | 2021 | Manych, Niccolò; Steckel, Jan Christoph; Jakob, Michael | Finance-based accounting of coal emissions | Environmental Research Letters |
| 94 | 2015 | Martí-Ballester, Carmen-Pilar | Can socially responsible investment for cleaner production improve the financial performance of Spanish pension plans? | Journal of Cleaner Production |
| 95 | 2021 | McDonnell, Clara; Rempel, Arthur; Gupta, Joyeeta | Climate action or distraction? Exploring investor initiatives and implications for unextractable fossil fuels | Energy Research & Social Science |
| 96 | 2021 | McGaughey, Ewan | Sustainable pensions, democratic governance, and EU law | European Journal of Social Security |
| 97 | 2019 | Mees, Bernard; Smith, Sherene A. | Corporate governance reform in Australia: A new institutional approach | British Journal of Management |
| 98 | 2020 | Mésonnier, Jean-Stéphane; Nguyen, Benoît | Showing off Cleaner Hands: Mandatory Climate-Related Disclosure by Financial Institutions and the Financing of Fossil Energy |  |
| 99 | 2019 | Mielke, Jahel | Signals for 2°C: the influence of policies, market factors and civil society actions on investment decisions for green infrastructure | Journal of Sustainable Finance & Investment |
| 100 | 2018 | Mielke, Jahel; Steudle, Gesine A. | Green Investment and Coordination Failure: An Investors' Perspective | Ecological Economics |
| 101 | 2019 | Neville, Kate J.; Cook, Jackie; Baka, Jennifer; Bakker, Karen; Weinthal, Erika S. | Can shareholder advocacy shape energy governance? The case of the US antifracking movement | Review of International Political Economy |
| 102 | 2022 | Ozili, Peterson K. | Green finance research around the world: a review of literature | International Journal of Green Economics |
| 103 | 2021 | Park, So Ra; Jang, Jae Young | The Impact of ESG Management on Investment Decision: Institutional Investors’ Perceptions of Country-Specific ESG Criteria | International Journal of Financial Studies |
| 104 | 2021 | Pearce, Prafula | Duty to Address Climate Change Litigation Risks for Australian Energy Companies—Policy and Governance Issues | Energies |
| 105 | 2019 | Peel, Jacqueline; Foerster, Anita; McDonnell, Brett; Osofsky, Hari M. | Governing the Energy Transition: The Role of Corporate Law Tools |  |
| 106 | 2013 | Peetz, David; Murray, Georgina | Financialization of corporate ownership and implications for the potential for climate action | Institutional Investors’ Power to Change Corporate Behavior: International Perspectives |
| 107 | 2008 | Pfeifer, Stephanie; Sullivan, Rory | Public policy, institutional investors and climate change: a UK case-study | Climatic Change |
| 108 | 2021 | Plantinga, Auke; Scholtens, Bert | The financial impact of fossil fuel divestment | Climate Policy |
| 109 | 2020 | Polzin, Friedemann; Sanders, Mark | How to finance the transition to low-carbon energy in Europe? | Energy Policy |
| 110 | 2019 | Puri, P. | Green but not enough: Sustainability in Canadian corporate governance | The Cambridge Handbook of Corporate Law, Corporate Governance and Sustainability |
| 111 | 2020 | Rempel, Arthur; Gupta, Joyeeta | Conflicting commitments? Examining pension funds, fossil fuel assets and climate policy in the organisation for economic co-operation and development (OECD) | Energy Research & Social Science |
| 112 | 2021 | Rempel, Arthur; Gupta, Joyeeta | Equitable, effective, and feasible approaches for a prospective fossil fuel transition | WIREs Climate Change |
| 113 | 2017 | Rezec, Michael; Scholtens, Bert | Financing energy transformation: The role of renewable energy equity indices | International Journal of Green Energy |
| 114 | 2019 | Rissman, Paul; Kearney, Diana | Rise of the Shadow ESG Regulators: Investment Advisers, Sustainability Accounting, and Their Effects on Corporate Social Responsibility | Environmental Law Reporter News & Analysis |
| 115 | 2022 | Ritz, Robert A. | Linking Executive Compensation to Climate Performance | California Management Review |
| 116 | 2022 | Rohleder, Martin; Wilkens, Marco; Zink, Jonas | The effects of mutual fund decarbonization on stock prices and carbon emissions | Journal of Banking & Finance |
| 117 | 2021 | Roncalli, Théo; Guenedal, Théo Le; Lepetit, Frédéric; Roncalli, Thierry; Sekine, Takaya | The Market Measure of Carbon Risk and its Impact on the Minimum Variance Portfolio | The Journal of Portfolio Management |
| 118 | 2021 | Saeudy, Mohamed; Atkins, Jill; Barone, Elisabetta A.V. | Interpreting banks’ sustainability initiatives as reputational risk management and mechanisms for coping, re-embedding and rebuilding societal trust | Qualitative Research in Financial Markets |
| 119 | 2022 | Sagbakken, Siri Tronslien; Zhang, Dan | European sin stocks | Journal of Asset Management |
| 120 | 2018 | Salisbury, Neil; Khvatsky, Jenya | Using Smart Algorithms, Machine Learning, and Blockchain Technology to Streamline and Accelerate Dealflow in Climate Finance | Transforming Climate Finance and Green Investment with Blockchains |
| 121 | 2021 | Sangiorgi, Ivan; Schopohl, Lisa | Why do institutional investors buy green bonds: Evidence from a survey of European asset managers | International Review of Financial Analysis |
| 122 | 2015 | Sarang, Surbhi | Combating Climate Change through a Duty to Divest | Columbia Journal of Law and Social Problems |
| 123 | 2021 | Schneider, Frederick; Gogolewska, Julia; Ahrend, Klaus-Michael; Hohendorf, Gerrit; Schneider, Gerhard; Busse, Reinhard; Schulz, Christian M. | Do private German health insurers invest their capital reserves of€ 353 billion according to environmental, social and governance criteria? | Journal of Medical Ethics |
| 124 | 2015 | Schneider, Nancy | Revisiting Divestment | Hastings Law Journal |
| 125 | 2020 | Scott, Inara | The Trouble with Boycotts: Can Fossil Fuel Divest Campaigns Be Prohibited? | American Business Law Journal |
| 126 | 2015 | Siew, Renard YJ | A review of corporate sustainability reporting tools (SRTs) | Journal of environmental management |
| 127 | 2011 | Solomon, Jill F.; Solomon, Aris; Norton, Simon D.; Joseph, Nathan L. | Private climate change reporting: an emerging discourse of risk and opportunity? | Accounting, Auditing & Accountability Journal |
| 128 | 2011 | Sørensen, Ole Beier; Pfeifer, Stephanie | Climate change issues in fund investment practices | International Social Security Review |
| 129 | 2021 | Strakodonskaya, Liudmila | How fiduciary duty law incentivises investors to manage sustainability risks | European Journal of Social Security |
| 130 | 2020 | Strampelli, Giovanni | Can BlackRock Save the Planet? The Institutional Investors' Role in Stakeholder Capitalism | Harvard Business Law Review Online |
| 131 | 2021 | Strauß, Nadine | Framing Sustainable Finance: A Critical Analysis of Op-eds in the Financial Times | International Journal of Business Communication |
| 132 | 2018 | Studart, Rogerio; Gallagher, Kevin | Guaranteeing sustainable infrastructure | International Economics |
| 133 | 2008 | Sullivan, R.; Pfeifer, S. | The evolution of UK institutional investor interest in climate change | Corporate Responses to Climate Change: Achieving Emissions Reductions through Regulation, Self-regulation and Economic Incentives |
| 134 | 2012 | Sullivan, Rory; Gouldson, Andy | Does voluntary carbon reporting meet investors’ needs? | Journal of Cleaner Production |
| 135 | 2022 | Taylor, Nick | ‘Making financial sense of the future’: actuaries and the management of climate-related financial risk | New Political Economy |
| 136 | 2020 | Taylor, Zac J. | The real estate risk fix: Residential insurance-linked securitization in the Florida metropolis | Environment and Planning A: Economy and Space |
| 137 | 2019 | Thomä, Jakob; Hayne, Michael; Hagedorn, Nikolaus; Murray, Clare; Grattage, Rebecca | The alignment of global equity and corporate bonds markets with the Paris Agreement: A new accounting framework | Journal of Applied Accounting Research |
| 138 | 2021 | Thomä, Jakob; Murray, Clare; Jerosch-Herold, Vincent; Magdanz, Janina | Do you manage what you measure? Investor views on the question of climate actions with empirical results from the Swiss pension fund and insurance sector | Journal of Sustainable Finance & Investment |
| 139 | 2018 | Trinks, A.; Scholtens, B.; Mulder, M.; Dam, L. | Fossil Fuel Divestment and Portfolio Performance | Ecological Economics |
| 140 | 2019 | Urban, Michael A.; Wójcik, Dariusz | Dirty Banking: Probing the Gap in Sustainable Finance | Sustainability |
| 141 | 2020 | van de Putte, Alexander; Campbell-Holt, Akshu, Littlejohn, George | Financing the Sustainable Energy Transition | The Geopolitics of the Global Energy Transition |
| 142 | 2020 | van der Ploeg, Frederick; Rezai, Armon | Stranded Assets in the Transition to a Carbon-Free Economy | Annual Review of Resource Economics |
| 143 | 2020 | Velte, Patrick; Obermann, Jörn | Compensation-related institutional investor activism – a literature review and integrated analysis of sustainability aspects | Journal of Global Responsibility |
| 144 | 2018 | Vörösmarty, C. J.; Osuna, V. Rodríguez; Koehler, D. A.; Klop, P.; Spengler, J. D.; Buonocore, J. J.; Cak, A. D.; Tessler, Z. D.; Corsi, F.; Green, P. A.; Sánchez, R. | Scientifically assess impacts of sustainable investments | Science |
| 145 | 2019 | Wasim, Roshaan | Corporate (non) disclosure of climate change information | Columbia Law Review |
| 146 | 2018 | Wirth, Eszter | The Norwegian Government Pension Fund Global, Corporate Social Responsibility and Climate Change | Revista: Revista de Economia Mundial, Periodo: 4, Volumen: , Número: 48, Página inicial: 179, Página final: 198 |
| 147 | 2011 | Woods, C. | Funding Climate Change: how pension fund fiduciary duty masks trustee inertia and short-termism. | Corporate Governance Failures: The Role of Institutional Investors in the Global Financial Crisis |
| 148 | 2013 | Wright, C. | Global Finance and the Environment | The Handbook of Global Climate and Environment Policy |
| 149 | 2021 | Yoshino, Naoyuki; Taghizadeh-Hesary, Farhad; Otsuka, Miyu | Covid-19 and Optimal Portfolio Selection for Investment in Sustainable Development Goals | Finance Research Letters |
| 150 | 2020 | Yunus, Somaiya; Elijido-Ten, Evangeline O.; Abhayawansa, Subhash | Impact of stakeholder pressure on the adoption of carbon management strategies: Evidence from Australia | Sustainability Accounting, Management and Policy Journal |
| 151 | 2022 | Zeidan, Rodrigo | Why don't asset managers accelerate ESG investing? A sentiment analysis based on 13,000 messages from finance professionals | Business Strategy and the Environment |
| 152 | 2019 | Zhang, Dayong, Zhang, Zhiwei, Managi, Shunsuke | A bibliometric analysis on green finance: Current status, development, and future directions | Finance Research Letters |
| 153 | 2022 | Zhang, Yiping; Weber, Olaf | Investors’ Moral and Financial Concerns—Ethical and Financial Divestment in the Fossil Fuel Industry | Sustainability |

1. PhD Researcher at the University of Amsterdam’s Governance and Inclusive Development Research Group, Department of Geography, Planning and International Development, Nieuwe Achtergracht 166, Amsterdam 1018WV, The Netherlands. ORCID: 0000-0003-1844-2977 [↑](#footnote-ref-1)
2. Professor of Environment and Development in the Global South, Amsterdam Institute for Social Science Research, University of Amsterdam; and IHE-Delft Institute for Water Education. ORCID: 0000-0003-1424-2660 [↑](#footnote-ref-2)
